# Prevalence and epidemiology of stroke in patients with multiple sclerosis: a systematic review and meta-analysis

**DOI:** 10.1007/s00415-024-12331-2

**Published:** 2024-04-04

**Authors:** Maria-Ioanna Stefanou, Vasileios Giannopapas, Dimitrios K. Kitsos, Maria Chondrogianni, Aikaterini Theodorou, Maria Kosmidou, Pinelopi Vlotinou, Christos Bakirtzis, Elizabeth Andreadou, John S. Tzartos, Sotirios Giannopoulos, Georgios Tsivgoulis

**Affiliations:** 1 Second Department of Neurology, School of Medicine, “Attikon” University Hospital, National and Kapodistrian University of Athens, Rimini 1, Chaidari, 12462 Athens, Greece; 2 Faculty of Medicine, Department of Internal Medicine, School of Health Sciences, University of Ioannina, Ioannina, Greece; 3 Department of Occupational Therapy, School of Health and Welfare Sciences, University of West Attica, Athens, Greece; 4 Second Department of Neurology and the MS Center, AHEPA University Hospital, Central Macedonia, Thessaloniki, Greece; 5 School of Medicine, First Department of Neurology, National and Kapodistrian University of Athens, “Eginition” University Hospital, Athens, Greece

**Keywords:** Multiple sclerosis, Stroke, Cardiovascular risk factors, Intracerebral haemorrhage, Cerebrovascular disease

## Abstract

**Background:**

Epidemiological data are sparse regarding the risk of stroke in patients with multiple sclerosis (MS).

**Objective:**

To estimate the following: (1) the pooled prevalence of all-cause stroke, acute ischaemic stroke (AIS) and intracerebral haemorrhage (ICH) in MS patients; (2) the relative risk for all-cause stroke, AIS and ICH in MS patients compared to the general population; (3) associations between patient characteristics and the risk for AIS and ICH in MS patients.

**Methods:**

Systematic review and meta-analysis of registry-based and cohort studies.

**Results:**

Thirteen observational studies comprising 146,381 MS patients were included. The pooled prevalence of all-cause stroke was 2.7% (95% confidence interval [CI] 1.3–4.6%), with the relative risk of all-cause stroke being higher in MS patients compared to the general population (RR: 2.55; 95% CI 1.97–3.29). Subgroup analyses *per* stroke subtype revealed a pooled AIS prevalence of 2.1% (95% CI 0.8–4.1%) and a pooled ICH prevalence of 0.6% (95% CI 0.2–1.2%). Compared to the general population, patients with MS were found to harbour an increased risk for AIS (RR: 2.79; 95% CI 2.27–3.41) and ICH (RR: 2.31; 95% CI 1.04–5.11), respectively. The pooled prevalence of cardiovascular risk factors in MS patients was 11.5% (95% CI 2.9–24.7%) for dyslipidaemia, 18.2% (95% CI 5.9–35.3%) for hypertension and 5.4% (95% CI 2.1–10.2%) for diabetes. In meta-regression, age was negatively associated with AIS risk (β =  – .03, *p* = 0.04), with a 1-year increase in age resulting in a significant 3% (95%CI 0–5) attenuation of the risk of AIS.

**Conclusion:**

The findings of the present meta-analysis indicate that MS is associated with an increased risk for ischaemic and haemorrhagic stroke. Future well-designed epidemiological studies are warranted to corroborate the robustness of the present findings in the MS population.

**Supplementary Information:**

The online version contains supplementary material available at 10.1007/s00415-024-12331-2.

## Introduction

Multiple sclerosis (MS) comprises a chronic inflammatory demyelinating disease of the human central nervous system, which (despite the recent tremendous therapeutic advances) still ranks as a leading cause of neurological disability among young and middle-aged adults worldwide [1]. With growing evidence, it has become apparent that the epidemiological landscape of MS continues to evolve, with population-specific genetic and environmental factors propelling changes in MS epidemiological metrics [2, 3]. Since the global disease burden of MS is rising, epidemiological studies disclose an exponential increase in the prevalence and incidence of MS across several geographic regions. These findings have largely been attributed to (i) improved survival of patients with MS, (ii) earlier MS diagnosis and (iii) shift in gene–environment interplays that exacerbate MS [2, 3].

On the other hand, disability accrual in MS has been increasingly acknowledged as not solely mediated by relapse-associated worsening, but also by disease progression independent of relapse activity [4]. With respect to the latter, it remains to date equivocal whether the accumulation of disability is strictly mediated by disease-specific immunological processes. An alternative hypothesis postulates that sustained blood–brain barrier (BBB) disruption, vascular changes and hypoxic cascades may partly contribute to relapse-independent neurodegeneration [5, 6]. In fact, epidemiological studies indicate that the patients with MS harbour an increased risk for vascular comorbidities and cerebrovascular diseases that may account for enhanced neurodegeneration and disability progression [7]. Beyond the epidemiological link, large-scale genetic and basic research studies have also recently suggested that neurovascular dysfunction may comprise a pathophysiologically intrinsic, albeit underrecognized, facet of MS [8]. The extent to which MS may correlate with cerebrovascular disease remains to be established.

In view of the former considerations, the aim of the present systematic review and meta-analysis was threefold. First, we sought to estimate the prevalence of all-cause stroke, acute ischaemic stroke (AIS) and intracerebral haemorrhage (ICH) in MS patients. Second, we attempted to examine the relative risk for all-cause stroke, AIS and ICH in patients with MS compared to the general population. Third, we aimed to investigate vascular comorbidities and patient characteristics associated with the AIS and ICH risk in MS patients.

## Methods

### Standard protocol approvals and registrations

The protocol for the present systematic review and meta-analysis has been pre-registered to the Open Search Foundation (OSF) (Registration: osf.io/7djhf). The updated Preferred Reporting Items for Systematic Reviews and Meta-Analyses (PRISMA) guidelines [9] have been employed for reporting, while reporting also adheres to the Meta-analysis of Observational Studies in Epidemiology (MOOSE) proposal [10]. Ethical board approval and individual written informed consent were not required for the present study as per the study design (systematic review and meta-analysis).

### Data sources, searches and study selection

A systematic literature search was independently performed by two reviewers (MIS, VG) to identify eligible studies that reported on AIS or ICH in MS patients. MEDLINE, Cochrane Library and SCOPUS databases were searched by applying search strings comprising the following search terms: “multiple sclerosis”, “stroke”, “intracranial haemorrhage”, “small vessel disease”, “vascular risk”, “vascular comorbidities” and “cardiovascular risk”. The full search algorithms that were used in MEDLINE, Cochrane Library and SCOPUS databases have been provided in the Supplement. The search spanned from each electronic database’s inception to 21 January 2024. To ascertain the comprehensiveness of the bibliography, reference lists of published articles fulfilling our inclusion criteria were searched manually.

Clinical trials, population-based studies or registries, along with observational cohort studies that reported on AIS or ICH in MS patients, were eligible for inclusion. Patients diagnosed with MS were considered eligible for inclusion, provided that the patient’s diagnosis was either with relapsing–remitting MS (RRMS), primary progressive MS (PPMS) or secondary progressive MS (SPMS). Per study protocol, studies were excluded if: (1) MS/AIS/ICH diagnoses were uncertain according to our pre-defined inclusion criteria; (2) reported outcomes were not aligned with our inclusion criteria; (3) and they were case reports, case series, narrative and systematic reviews, commentaries, pre-prints or non-peer reviewed studies and conference abstracts. In case that studies had overlapping data, we retained the study with the largest dataset. All retrieved studies were assessed by two reviewers (MIS, VG) independently, and any disagreements between reviewers were resolved after discussion with a third tie-breaking evaluator (GT).

### Quality control, bias assessment and data extraction

For relevant domains of each included study, the risk of bias was assessed using the Risk Of Bias In Non-randomized Studies of Interventions (ROBINS-I) tool [11]. Two independent reviewers (MIS, VG) performed quality control and bias assessment, and consensus after discussion with the corresponding author (SG) was reached in case of disagreement. For further analyses, data including author names, date of publication, study design, country, event type (i.e. all-cause stroke, AIS or ICH) and patient characteristics were extracted from individual studies in structured reports.

### Outcomes

We performed an aggregate data meta-analysis by including identified population-based studies or registries, and observational cohort studies.

The pre-defined primary outcome measures of the present meta-analysis were twofold: (i) the pooled prevalence of all-cause stroke, AIS and ICH in MS patients and (ii) the relative risk for all-cause stroke, AIS and ICH in MS patients compared to the general population. For relative risk assessment, only studies that provided estimates in both MS patients and the general population (i.e. controls) were considered. Secondary outcomes of interest comprised the prevalence of vascular comorbidities in patients with MS. Additionally, associations between demographic characteristics and MS-related characteristics and AIS or ICH diagnosis were assessed in the MS population. Sensitivity analyses were also performed after the exclusion of low-quality studies and after the exclusion of studies with the duration shorter than 10 years.

### Statistical analysis

For each dichotomous outcome of interest, the pooled prevalence with 95% confidence intervals (95% CI) was calculated for the aggregate meta-analysis, after the implementation of the Freeman–Tukey variance-stabilizing double arcsine transformation [12–14]. The random-effects model of meta-analysis (DerSimonian and Laird) was utilized for estimation of the pooled estimates [15]. Pairwise comparisons were conducted between MS patients and the general population and were reported using risk ratios (RRs) and corresponding 95% confidence intervals (95% CI). We used the Q test to assess subgroup differences [16]. Accordingly, the I^2^ and Cochran Q statistics were employed for heterogeneity assessment. With respect to the qualitative heterogeneity interpretation, I^2^ values > 50% and values > 75% were regarded to represent either substantial or considerable heterogeneity, respectively. The significance level was set at 0.1 for the Q statistic. Graphical assessment of publication bias was performed across individual studies when more than four studies were included for each primary outcome-of-interest analysis, we used a funnel and radial plot inspection and the Egger’s linear regression test accordingly [17], while the equivalent z test with a two-tailed p value < 0.05 was considered statistically significant for each pooled estimate. All statistical analyses and figure production were carried out using RStudio for Windows [R studio/R Meta package].

## Results

### Literature search and included studies

**Fig. 1:**
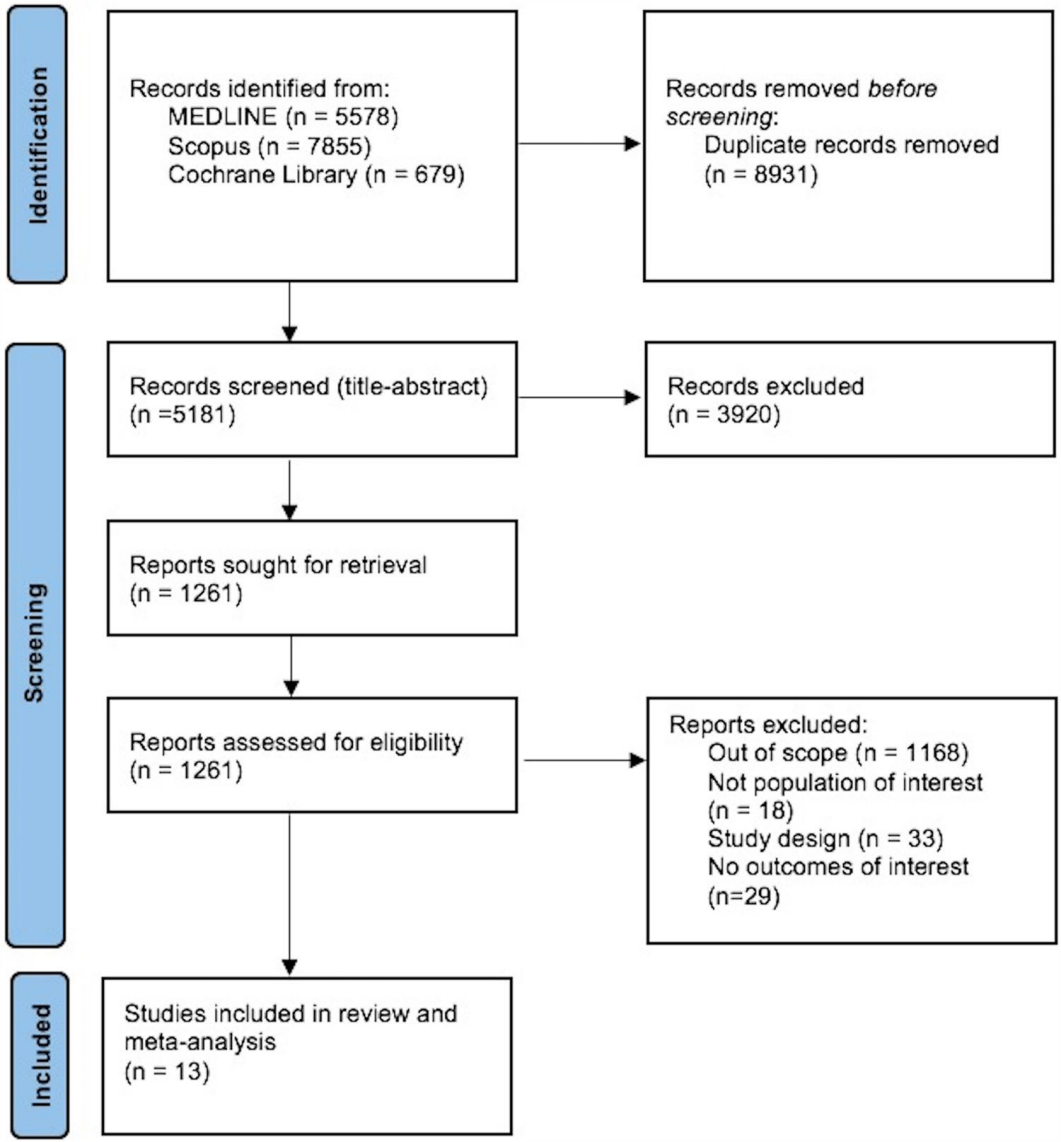
Prisma flow chart of included studies

The systematic database search yielded 14,112 records from MEDLINE, SCOPUS and Cochrane Library databases. After the exclusion of duplicates and articles that were out of scope, 1,261 records were considered eligible for inclusion and were assessed in full. After reading the full-text articles, 1,248 were further excluded (Supplement). Finally, we identified 13 eligible studies [18–30] for inclusion comprising a total of 146,381 MS patients. All retrieved studies were observational, and Table 1 summarizes their main characteristics, including country of origin, study type, population size and reported outcomes. In Fig. 1, the PRISMA flow chart of the meta-analysis is presented.

**Table 1:** Main characteristics of studies (*n* = 13) included in the systematic review

Study	Country	Study type	Sample				Type of stroke		
			MS stroke +	MS total	Control stroke +	Control stroke	All-cause stroke	AIS	ICH
Allen et al. 2008 [18]	USA	Registry	178	9949	268	19,898	+	+	+
Christiansen et al. 2010 [19]	Denmark	Cohort	38	13,963	49	66,407	+	+	
Lavela et al. 2012 [20]	USA	Cohort	80	1142	957	68,357	+		
Zöller et al. 2012 [21]	Sweden	Registry	88	10,384			+	+	+
Jadidi et al. 2013 [22]	Sweden	Registry	194	7664	728	66,214	+		
Tseng et al. 2014 [23]	Taiwan	Cohort	41	1174	44	4696	+	+	
Capcun et al. 2015 [24]	USA	Cohort	1880	15,684	2812	78,420	+	+	+
Thorman et al. 2016 [25]	Denmark	Registry	565	8947	1677	44,735	+		
Persson et al. 2017 [26]	UK/USA	Registry	88	12,132	456	123,612	+	+	
Zulfiqar et al. 2018 [27]	Mexico	Cohort	107	57,099			+		
Castelo-Branco et al. 2020 [28]	Sweden	Cohort	159	6602	518	61,828	+	+	
Tadic et al. 2020 [29]	Bosnia and Herzegovina	Cohort	1	100	2	50	+		
Cho et al. 2022 [30]	South Korea	Cohort	86	1541	171	7705	+	+	+

Abbreviations: MS: multiple sclerosis; AIS: acute ischaemic attack; ICH: intracerebral haemorrhage

### Quality control of included studies

The risk of bias of studies included in the present meta-analysis was assessed using the Risk Of Bias In Non-randomized Studies of Interventions (ROBINS-I) tool [11] and is presented in Supplementary Fig. 1. The majority of studies presented significant biases due to confounding (i.e. by not reporting key confounding variables such as MS subtypes, disability status or stroke characteristics), as well as biases in the classification of primary outcomes (i.e. diagnostic criteria not reported) (Table 2).

**Table 2:** Overview of analyses for primary and secondary outcomes

Variable	Analysis			Analysis		
	*N* of studies	Pooled estimates (95% CI)	I^2^, *p* for Cochran Q	*N* of studies	Pooled risk ratio (95% CI)	I^2^, *p* for Cochran Q
*Primary outcome*						
Stroke (all-cause)	13	2.7% (1.3–4.6)	100%, *p* = 0	11	2.55 (1.97–3.29)	96%, *p* < 0.01
AIS	8	2.1% (0.8–4.1)	100%, *p* = 0	7	2.79 (2.27–3.41)	84%, *p* < 0.01
ICH	4	0.6% (0.2–1.2)	98%, p < 0.01	3	2.31 (1.05–5.12)	94%, *p* < 0.01

Abbreviations: CI: confidence interval; AIS: acute ischaemic attack; ICH: intracerebral haemorrhage

### Quantitative analyses

#### Primary outcome

**Fig. 2:**
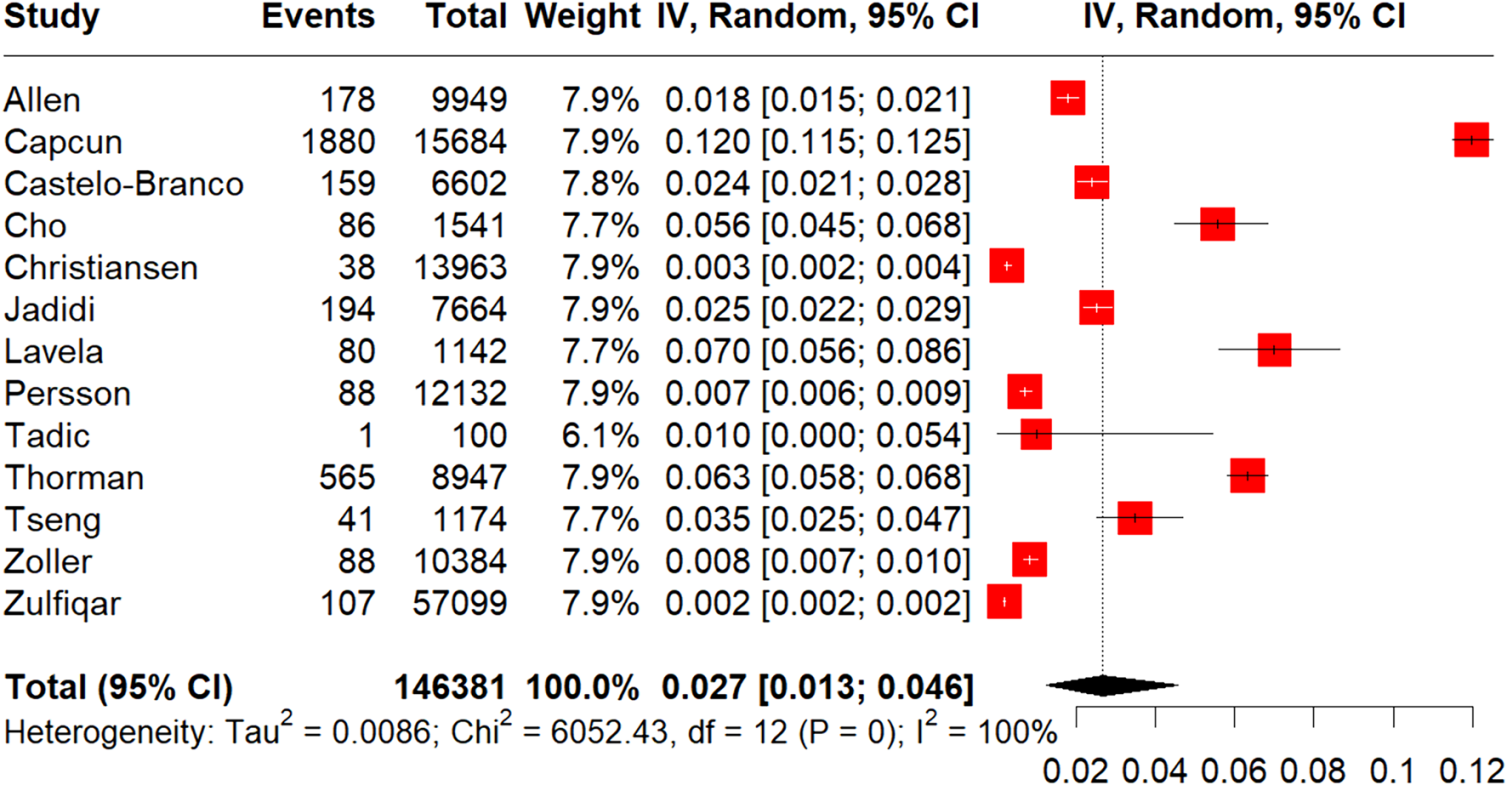
Pooled prevalence of all-cause stroke in MS patients

**Fig. 3:**
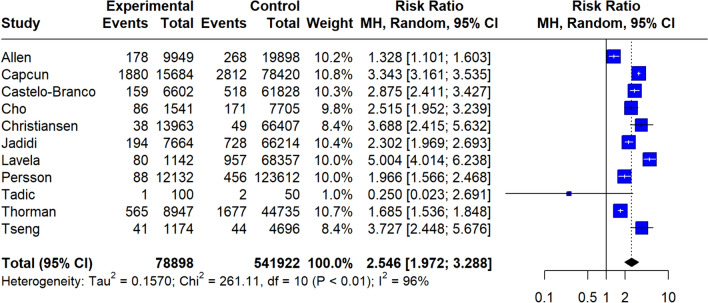
Risk ratio of all-cause stroke in the MS population compared to the general population

**Fig. 4:**
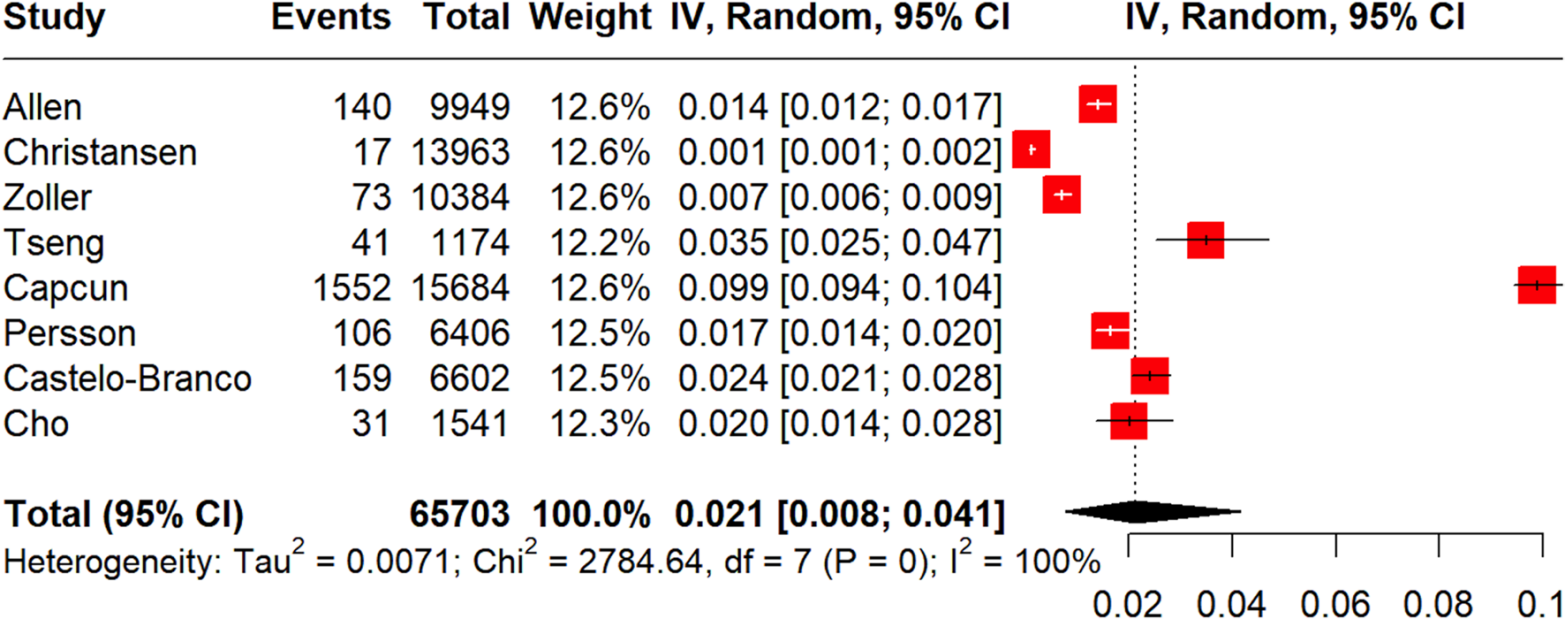
Pooled prevalence of acute ischaemic stroke (AIS) in the MS population

**Fig. 5:**
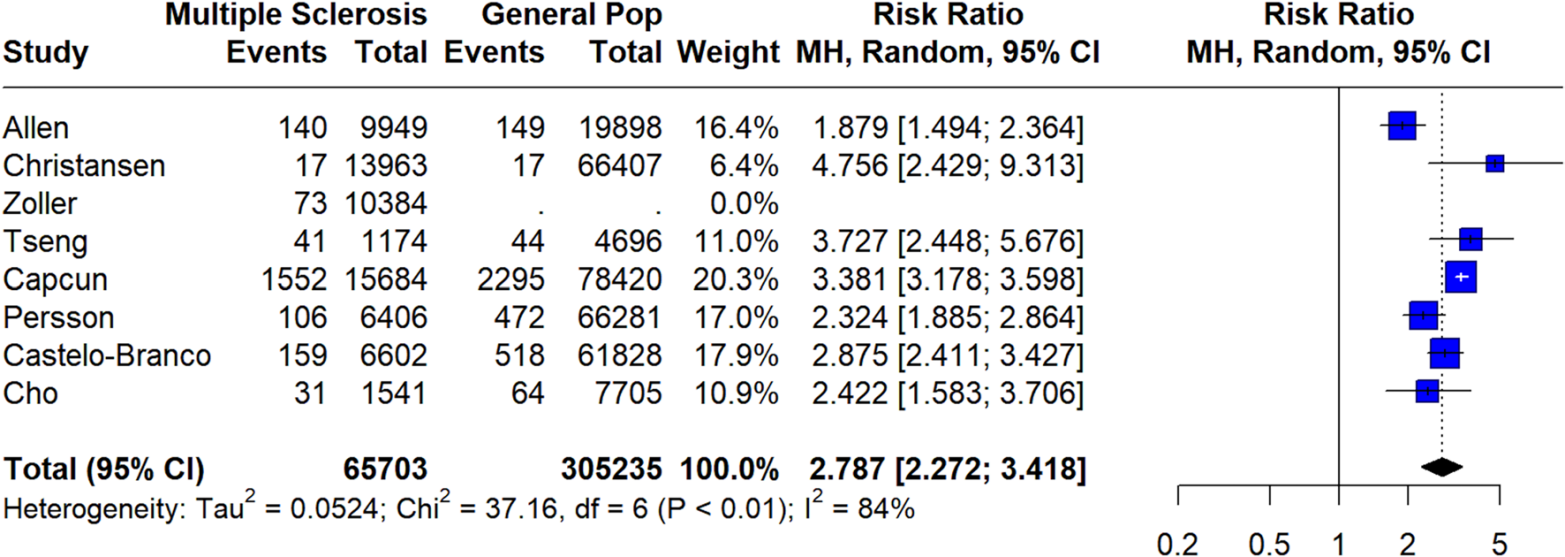
Risk ratio of acute ischaemic stroke (AIS) in the MS population compared to the general population

**Fig. 6:**
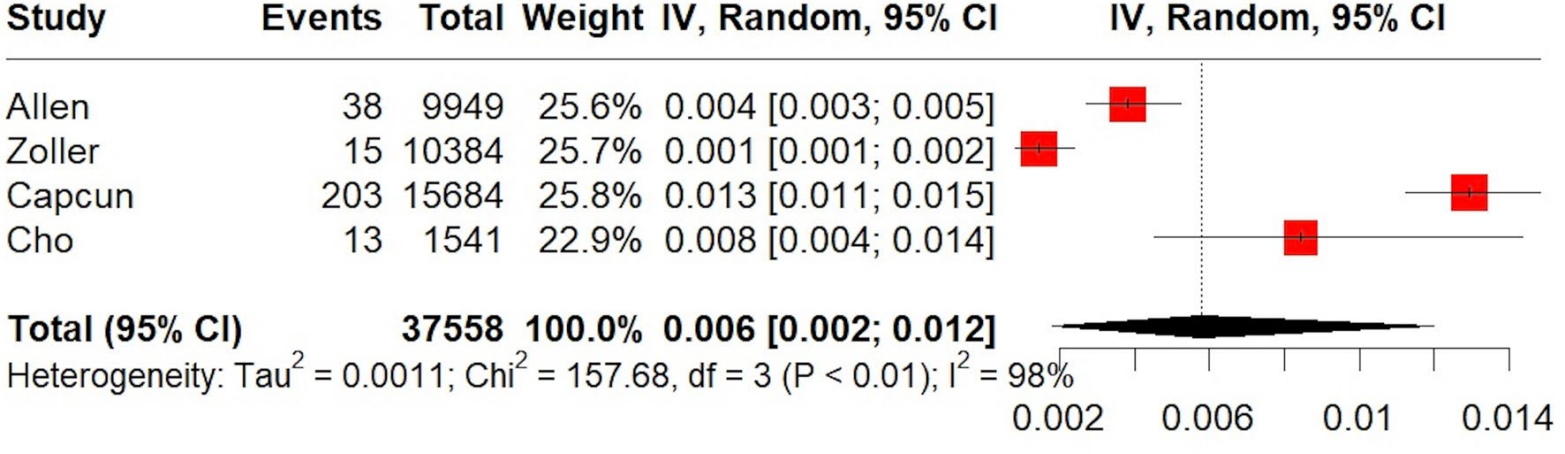
Pooled prevalence of intracerebral haemorrhage (ICH) in the MS population

**Fig. 7:**
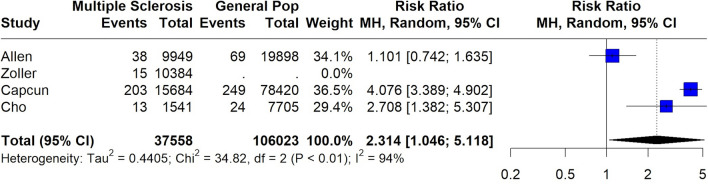
Risk ratio of intracerebral haemorrhage (ICH) in the MS population compared to the general population

A total of 146,381 MS patients were included in the meta-analysis. MS diagnosis was ascertained by the use of International Classification of Diseases (ICD) codes in all included studies, with the exception of the study by Tadic et al. [29] which reported that only patients with “definitive MS” were included. The pooled prevalence of all-cause stroke among MS patients was 2.7% (95% CI 1.3–4.6%;13 studies; *I*^2^ = 100%, *p* for Cochran Q = 0; Fig. 2) [18–30], with the relative risk of all-cause stroke being significantly higher in MS patients compared to the general population (RR: 2.55; 95% CI 1.97–3.29; 11 studies; I^2^ = 96%; *p* for Cochran Q < 0.01)[18–30] (Fig. 3). When the data were stratified according to stroke subtype, the pooled prevalence of AIS in MS patients was 2.1% (95% CI 0.8–4.1%; eight studies; *I*^2^ = 100%, *p* for Cochran Q = 0; Fig. 4) [18, 19, 21, 23, 24, 26, 28, 30]; the relative risk of AIS in MS patients compared to the general population was 2.79 (95% CI 2.28–3.42; seven studies; I^2^ = 84%; *p* for Cochran Q < 0.01; Fig. 5) [18, 19, 21, 23, 24, 26, 28, 30]. With respect to ICH, the pooled ICH prevalence in MS patients was 0.6% (95% CI 0.2–1.2%; four studies; I^2^ = 98%, *p* for Cochran Q < 0.01; Fig. 6) [18, 21, 24, 30]; the relative risk for ICH in patients with MS compared to the general population was 2.31 (95% CI 1.05–5.12; three studies; I^2^ = 94%; *p* for Cochran Q < 0.01; Fig. 7) [18, 21, 24, 30].

### Secondary outcomes

With respect to cardiovascular risk factors, the pooled prevalence of hypertension, diabetes and dyslipidaemia were assessed in MS patients. The pooled prevalence of hypertension was 18.2% (95% CI 5.9–35.3%; seven studies; I^2^ = 100%; *p* for Cochran Q = 0, Supplementary Fig. 2) in MS patients [20, 22–25, 29, 30]. The pooled prevalence of diabetes and dyslipidaemia was 5.4% (95% CI 2.1–10.2%; six studies; I^2^ = 99%; *p* for Cochran Q < 0.01; Supplementary Fig. 3) [19, 23, 24, 28–30] and 11.5% (95% CI 2.9–24.7%; six studies; *I*^2^ = 100%; *p* for Cochran Q = 0; Supplementary Fig. 4) [20, 23, 24, 28–30], respectively.

With respect to the association between characteristics of MS patients and the risk of stroke, a subgroup analysis was performed including only studies that reported the mean age of MS patients. In particular, meta-regression was possible for all-cause stroke and AIS patients, but not for individuals with ICH due to data unavailability. Meta-regression analysis revealed that age is not a significant predictor neither of all-cause stroke (*p* = 0.96), nor AIS (*p* = 0.57). No significant association was disclosed between age and the risk of all-cause stroke (*p* = 0.66). However, there was a statistically significant negative association between age and the risk of AIS (β =  – 0.03; 95% CI  – 0.06–0, *p* = 0.04), with a 1-year increase in age resulting in a significant 3% (95% CI 0–5) attenuation of AIS risk [18, 23, 24, 28] (Supplementary Fig. 5).

### Sensitivity analysis

Sensitivity analyses were subsequently performed to assess the prevalence and relative risk of all-cause stoke after the exclusion of low-quality studies [20, 29]. The pooled prevalence of all-cause stroke was 2.5% (95% CI 1.1–4.6%; 11 studies; *I*^2^ = 100%, *p* for Cochran Q = 0; Supplementary Fig. 6), while the relative risk for all-cause stroke between patients with MS and the general population was 2.41 (95% CI 1.91–3.04; nine studies; I^2^ = 96%, *p* for Cochran Q < 0.01; Supplementary Fig. 7). Furthermore, an additional analysis after the exclusion of studies with the duration shorter than 10 years [20, 22, 24, 28, 30] revealed a 2.1% pooled prevalence for all-cause stroke (95% CI 0.9–3.9%; seven studies; *I*^2^ = 100%; *p* for Cochran Q = 0; Supplementary Fig. 8) and a relative risk for all-cause stroke of 1.65 (95% CI 0.98–2.76; five studies; *I*^2^ = 98%, *p* for Cochran Q < 0.01; Supplementary Fig. 9). Sensitivity analyses stratified by stroke subtype could not be performed due to data unavailability.

### Publication bias

Funnel plots were employed to evaluate publication bias for the primary outcome of interest. Regarding all-cause stroke prevalence, moderate to high funnel plot asymmetry was uncovered (Supplementary Fig. 10) with a statistically significant Egger’s test (*p* < 0.01). Regarding the risk ratio of all-cause stroke in MS patients compared to the general population, funnel plot inspection revealed moderate asymmetry with a non-significant Egger’s test (*p* = 0.17) (Supplementary Fig. 11).

## Discussion

In the present systematic review and meta-analysis, the pooled prevalence of all-cause stroke among patients with MS was estimated at 2.7 cases per 100 patients (95%CI 1.3–4.6%), with the relative risk of all-cause stroke being more than twofold higher in patients with MS compared to the general population (RR:2.55; 95%CI 1.97–3.29). Additionally, an increased predisposition of patients with MS to ischaemic rather than haemorrhagic stroke was disclosed, as indicated by the higher cumulative prevalence of AIS of 2.1% (95%CI 0.8–4.1%) as opposed to the cumulative ICH prevalence of 0.6% (95%CI 0.2–1.2%). Accordingly, comparisons between patients with MS and the general population revealed that MS patients harbour an increased risk for AIS and ICH, respectively.

These findings are in accordance with the results of prior population-based cohorts and registries that ascertain an increased risk of MS patients for cerebrovascular and cardiovascular diseases [12, 31]. In particular, cardiovascular risk factors were documented with varying prevalence among patients with MS, with crude prevalence estimates for hypertension of 18.2% (95% CI 5.9–35.3%), diabetes: 5.4% (95% CI 2.1–10.2%) and dyslipidaemia: 11.5% (95% CI 2.9–24.7). Notably, vascular comorbidities in MS have been previously linked to (i) the sedentary lifestyle and immobility of MS patients [32, 33], (ii) the excess risk for psychiatric comorbidities [34], (iii) MS-related cardiac autonomic dysfunction[35] and (iv) shared genetic variants between MS and cerebrovascular diseases as revealed by large-scale genome-wide association studies [36, 37]. Notwithstanding the evidence of a heightened risk for vascular comorbidities, however, recent studies suggest that MS may comprise an independent risk factor for cerebrovascular disease (i.e. with the cerebrovascular risk in MS not fully accounted for by traditional vascular risk factors) [38, 39].

From a pathophysiological perspective, several MS-specific mechanisms have been implicated in incident cerebrovascular disease in MS patients. First, chronic inflammation has been linked to endothelial dysfunction and distinct arteriolar changes (i.e. arteriolosclerosis, periarteriolar space dilatation and hemosiderin deposition), which in turn precipitate micro-ischaemia and micro-haemorrhages within the cerebral white matter [6, 40]. Second, pathological studies comparing patients with MS to age-matched controls have disclosed a significant correlation between cerebral small vessel disease—but not large artery atherosclerosis—and MS [6]. Third, the use of certain disease-modifying therapies (DMTs) has been associated with cardiovascular complications, including cardiotoxicity, haemodynamic impairment, hypertensive derailment and increased risk for ICH (e.g. alemtuzumab) [41–44]. With respect to the latter, although we cannot exclude that surveillance and reporting biases may partly account for an overestimation of the crude ICH prevalence in patients with MS, it is striking that the recorded proportion of ischaemic over haemorrhagic stroke in patients with MS was aligned with to the global, epidemiological, age-standardized 3.5:1 ratio of ischaemic vs. haemorrhagic stroke [45]. To the best of our knowledge, the present meta-analysis is the first to provide evidence of an increased predisposition of patients with MS to ICH. With the established venocentric progression of MS lesions in mind, chronic cerebrospinal venous insufficiency and hampered cerebrospinal venous drainage may comprise additional pathways that independently confer an enhanced risk for cerebral microbleeds and ICH in patients with MS [46, 47].

In addition, we found a significant negative association between age and the risk of AIS in patients with MS, with a 1-year increase in age resulting in a significant 3% attenuation of the risk for AIS. These results are aligned with evidence from previous studies that indicate a time-dependent decrease in the risk of stroke in MS patients, but also in patients with other immune-mediated diseases (including ankylosing spondylitis, polymyalgia rheumatica, rheumatoid arthritis, systemic lupus erythematosus, Wegener’s granulomatosis, Crohn’s disease, ulcerative colitis, immune thrombocytopenic purpura, polymyositis/dermatomyositis and Sjögren’s syndrome) [21]. Several mechanisms may account for the previous findings, including (i) haemostatic imbalance (i.e. procoagulant upregulation, anticoagulant downregulation and fibrinolysis suppression) in the setting of untreated immune-mediated disease [48], (ii) pro-coagulatory effects of corticosteroids [49], and (iii) decreasing inflammatory activity and hence cardiovascular risk following DMT initiation [50]. Although data on DMTs were not available for meta-regression, it would be compelling for future studies to assess the potential association of DMTs with the risk of stroke in MS patients.

Taken together, the results of the present meta-analysis expand and strengthen the findings of previous research [51], by incorporating data from recently published studies [26–30], while providing prevalence estimates *per* stroke subtype and cardiovascular comorbidity in the MS population. Nonetheless, certain limitations should be acknowledged for an accurate interpretation of our findings. First, there was significant heterogeneity among included studies, both in terms of methodology and population characteristics, which may have confounded the pooled prevalence estimates. Nevertheless, sensitivity analyses after the exclusion of low-quality studies and studies with the duration shorter than 10 years did not affect the pooled estimates. Further epidemiological research is warranted to evaluate the generalizability of the present results. Second, all included studies were observational and may have suffered from selection or reporting biases, a fact that hinders robust inferences from noted associations. Third, only a limited number of studies provided data for meta-regression; importantly, disability parameters (i.e. Expanded Disability Status Scale—EDSS), stroke aetiology [52, 53], DMTs and MS subtypes were not systematically documented in included studies. Thus, further subgroup or sensitivity analyses could not be performed. Consequently, larger well-characterized cohorts and registries (i.e. providing individual patient or stratified data including EDSS, stroke aetiology, MS subtype and DMTs) are urgently required to delineate stroke characteristics in MS patients with the aim to unravel causal associations between MS and stroke. Fourth, residual confounding due to publication bias cannot be excluded; thus, replication of the present results is warranted in the context of large, multicentre observational and epidemiological studies.

In conclusion, the findings of the present meta-analysis indicate a positive association between MS and risk of all-cause stroke, AIS and ICH. Given the aforementioned methodological limitations, including the high heterogeneity in reported outcomes, potential presence of publication bias and overall moderate quality of studies included in the present meta-analysis, the future well-designed epidemiological research is required to corroborate our findings. Beyond traditional cardiovascular risk factors that confer a heightened risk of stroke in MS patients, the evidence of a “paradoxical” attenuation of the risk of stroke with increasing patient age may be aligned with the hypothesis of disease activity comprising an independent risk factor for cerebrovascular disease in MS. While further research is required to elaborate the pathophysiological associations behind this correlation, clinicians should recognize the elevated stroke risk and prioritize targeted stroke prevention strategies in the MS patient population to reduce stroke burden and improve patient outcomes.

### Supplementary Information

Below is the link to the electronic supplementary material.

Supplementary file1 (DOCX 616 KB)

## Data Availability

All data analysed in the present study have been included in the present article and its supplementary material.
