# Supplementary material for: Prevalence and epidemiology of stroke in patients with multiple sclerosis: a systematic review and meta-analysis
Source: J Neurol. 2024 Apr 4;271(7):4075–85. doi: 10.1007/s00415-024-12331-2 (PMC11233381; doi:10.1007/s00415-024-12331-2)

**Supplementary materials**

**Supplementary Figures:1-11**

Supplementary Figure 1: Traffic light plot Robins-I

Supplementary Figure 2: Prevalence of hypertension in the included MS population

Supplementary Figure 3: Prevalence of diabetes in the included MS population

Supplementary Figure 4: Prevalence of dyslipidemia in the included MS population

Supplementary Figure 5: Meta-regression bubble plot: association of age with risk ratio of acute ischemic stroke (AIS)

Supplementary Figure 6: Pooled prevalence of all-cause stroke in MS patients in sensitivity analysis after exclusion of low-quality studies

Supplementary Figure 7: Relative risk for all-cause stroke in the MS population compared to the general population in sensitivity analysis after exclusion of low-quality studies

Supplementary Figure 8: Pooled prevalence of all-cause stroke in MS patients in sensitivity analysis after exclusion of studies with duration shorter than 10 years

Supplementary Figure 9: Relative risk for all-cause stroke in the MS population compared to the general population in sensitivity analysis after exclusion of studies with duration shorter than 10 years

Supplementary Figure 10: Funnel Plot of all-cause stroke prevalence in the MS population

Supplementary Figure 11: Funnel Plot of the relative risk for all-cause stroke in the MS population compared to the general population

**eMethods**

**Complete search algorithm used in MEDLINE search.**

(multiple sclerosis) AND ((stroke) OR (intracranial hemorrhage) OR (small vessel disease) OR (vascular risk) OR (vascular comorbidities) OR (cardiovascular risk))

**Complete search algorithm used in SCOPUS search.**

TITLE-ABS-KEY ( multiple AND sclerosis AND ( intracranial AND hemorrhage ) OR ( stroke ) OR ( small AND vessel AND disease ) OR ( vascular AND risk ) OR ( vascular AND comorbidities ) OR ( cardiovascular AND risk ) )

**Complete search algorithm used in Cochrane Library.**

(multiple sclerosis) AND ((stroke) OR (intracranial hemorrhage) or (small vessel disease) OR (vascular risk) OR (vascular comorbidities) OR (cardiovascular risk))

**Excluded studies, with reasons, can be found at the OSF registry :** osf.io/7djhf

Supplementary Figure 1:Trafic Light Plot (Robins-I tool)


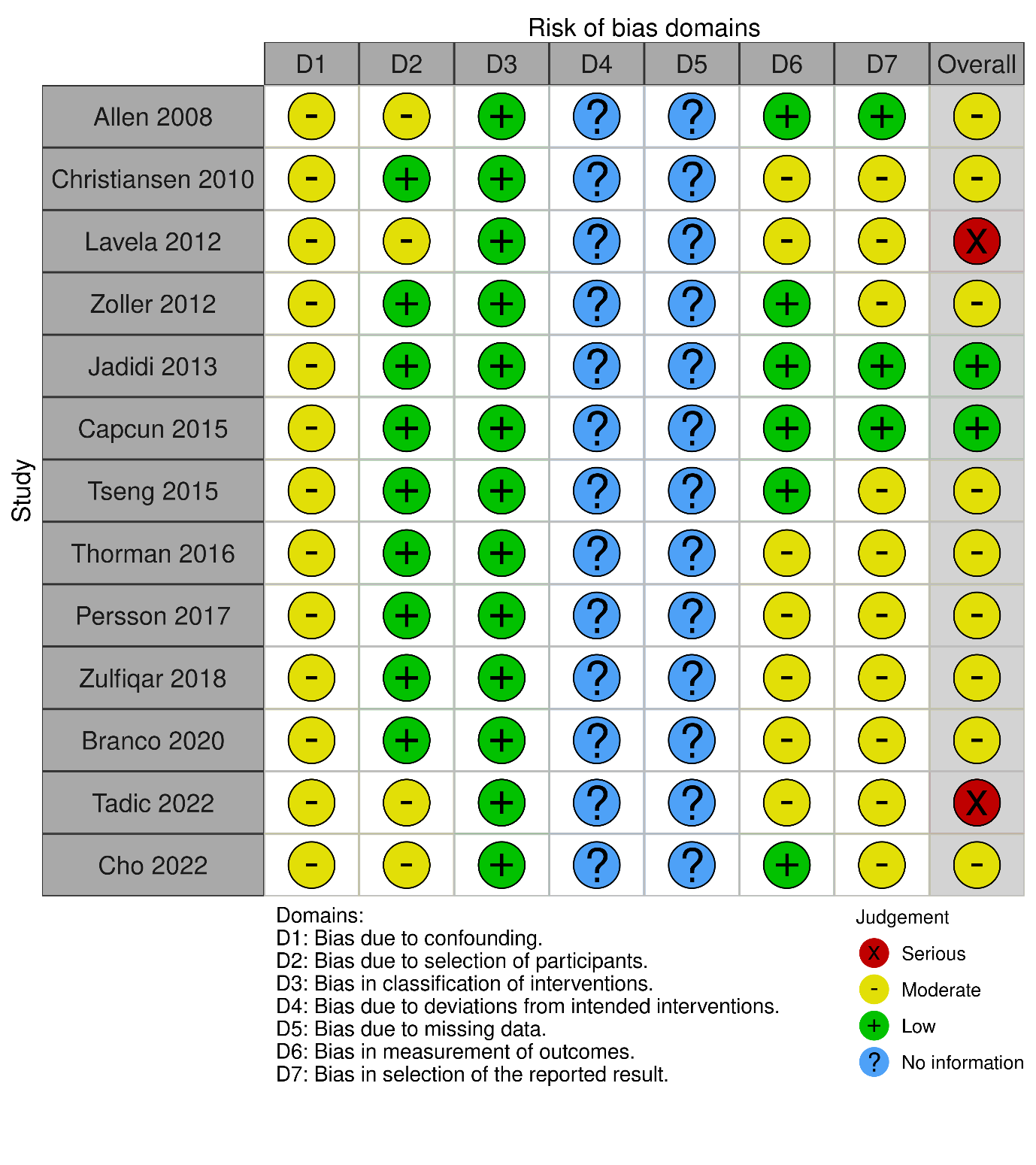


Supplementary Figure 2: Prevalence of hypertension in the included MS population


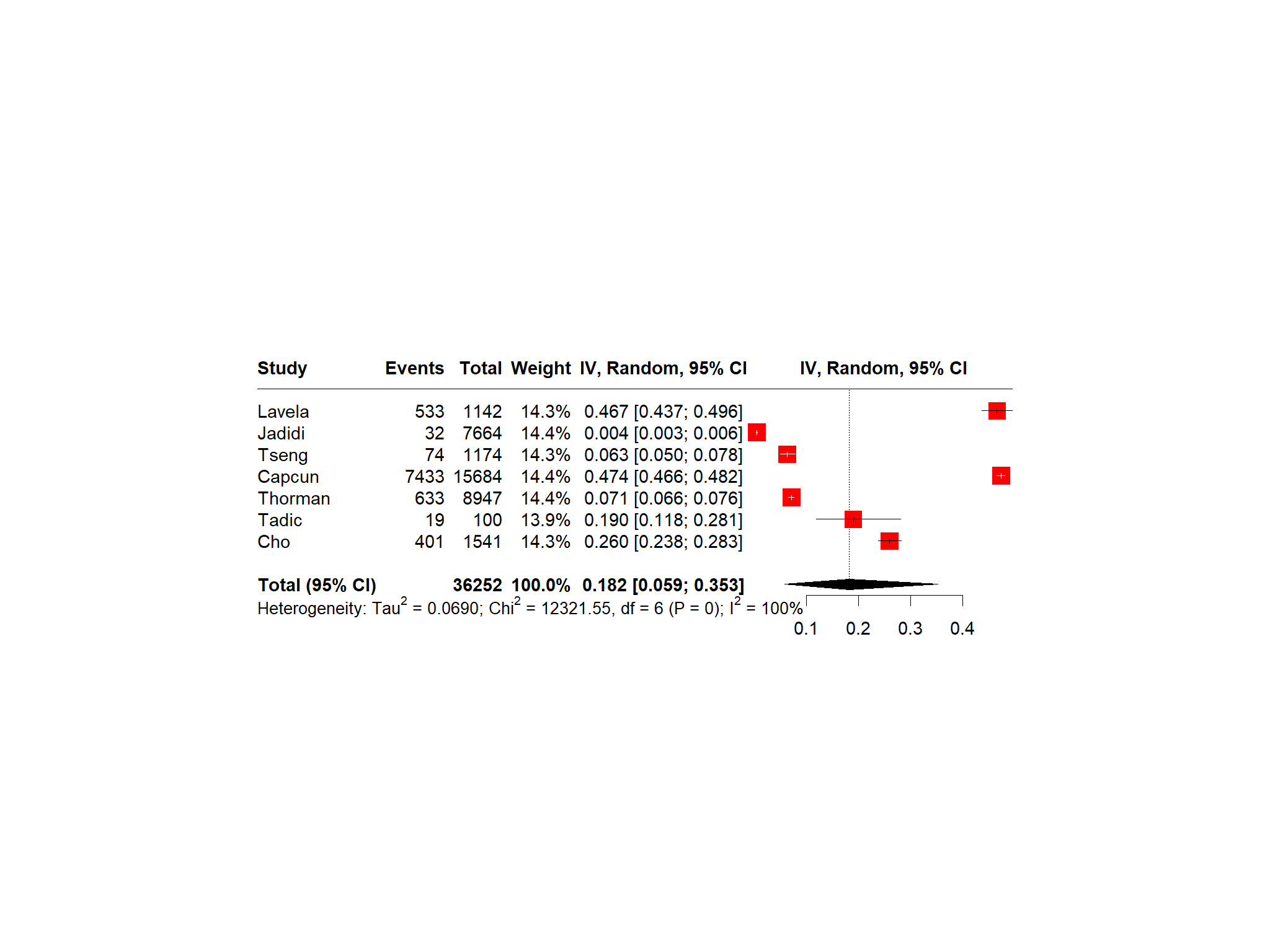


Supplementary Figure 3: Prevalence of diabetes in the included MS population


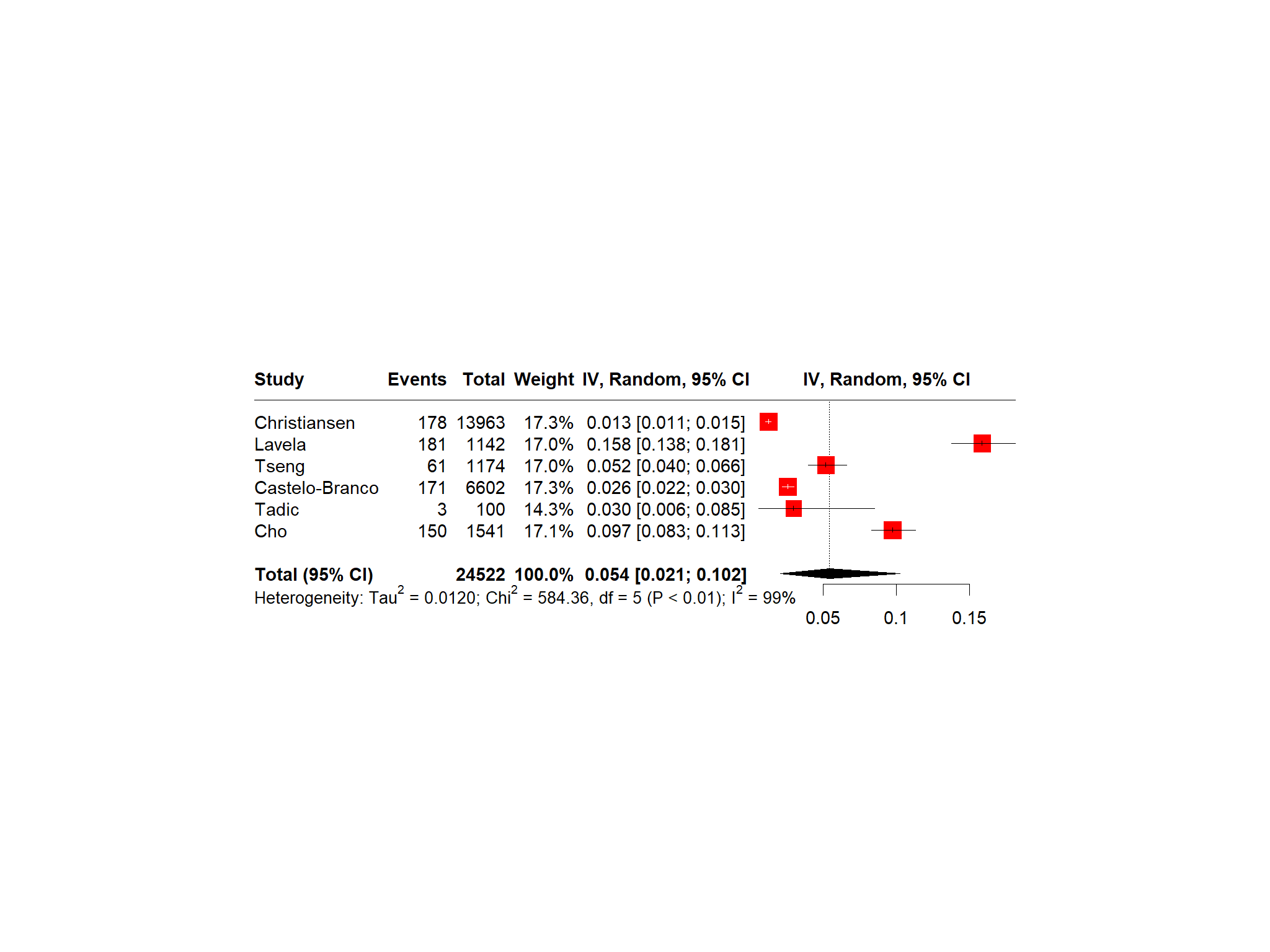


Supplementary Figure 4: Prevalence of dyslipidemia in the included MS population


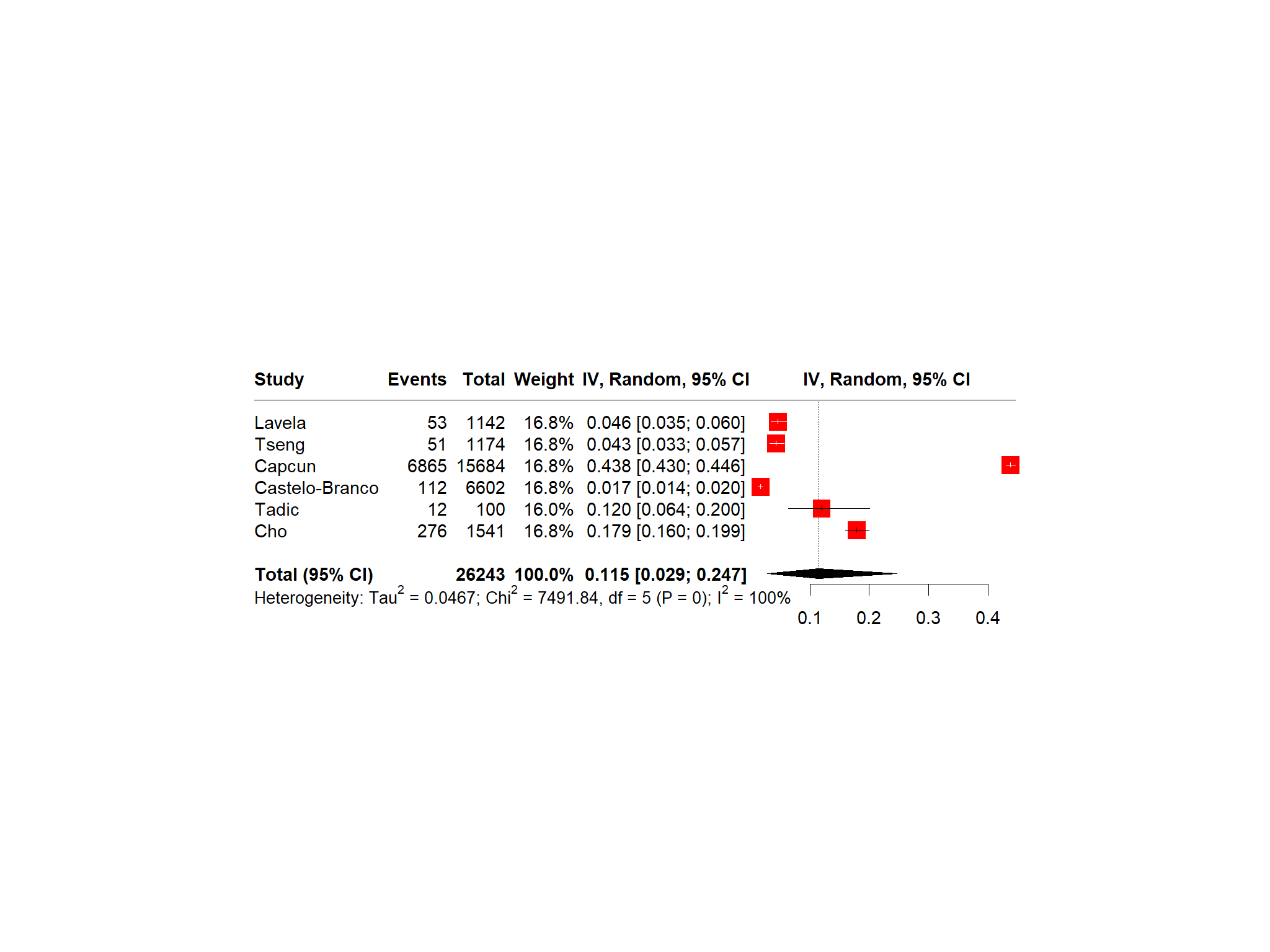


Supplementary Figure 5: Meta-regression bubble plot: association of age with risk ratio of acute ischemic stroke (AIS)


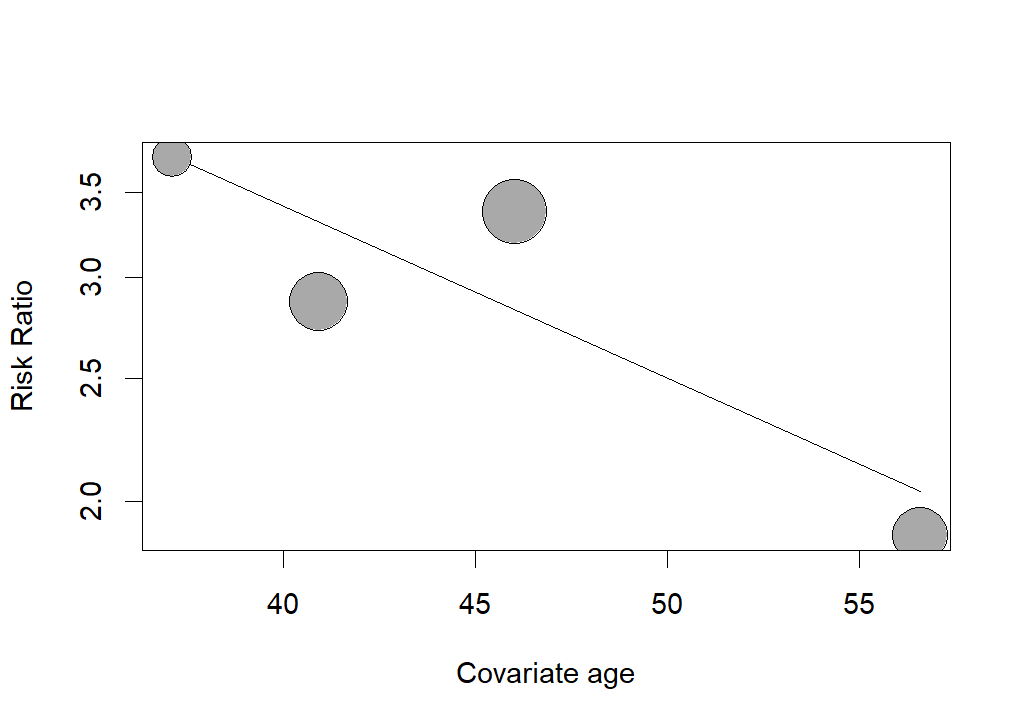


Supplementary Figure 6: Pooled prevalence of all-cause stroke in MS patients in sensitivity analysis after exclusion of low-quality studies


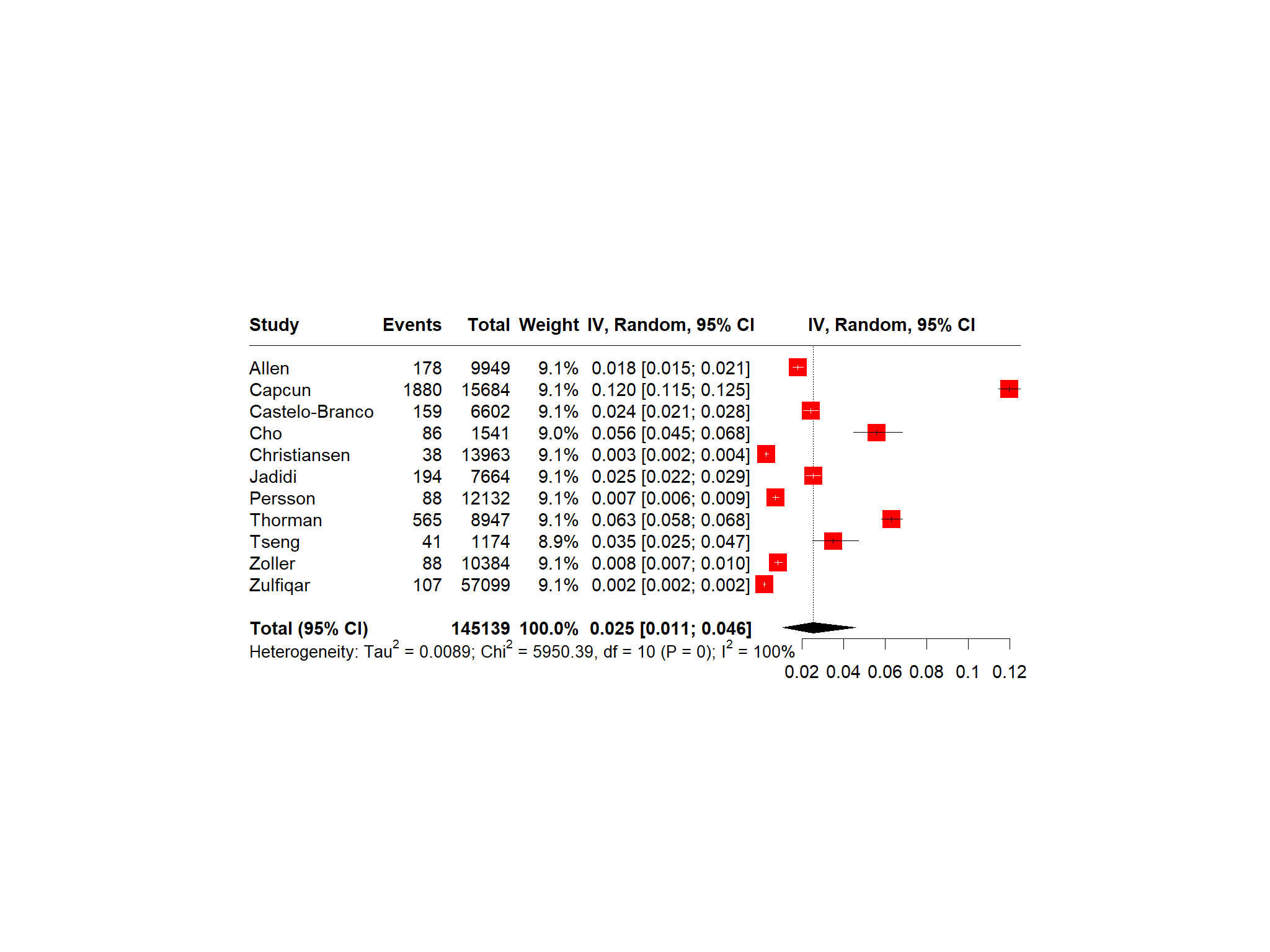


Supplementary Figure 7: Relative risk for all-cause stroke in the MS population compared to the general population in sensitivity analysis after exclusion of low-quality studies


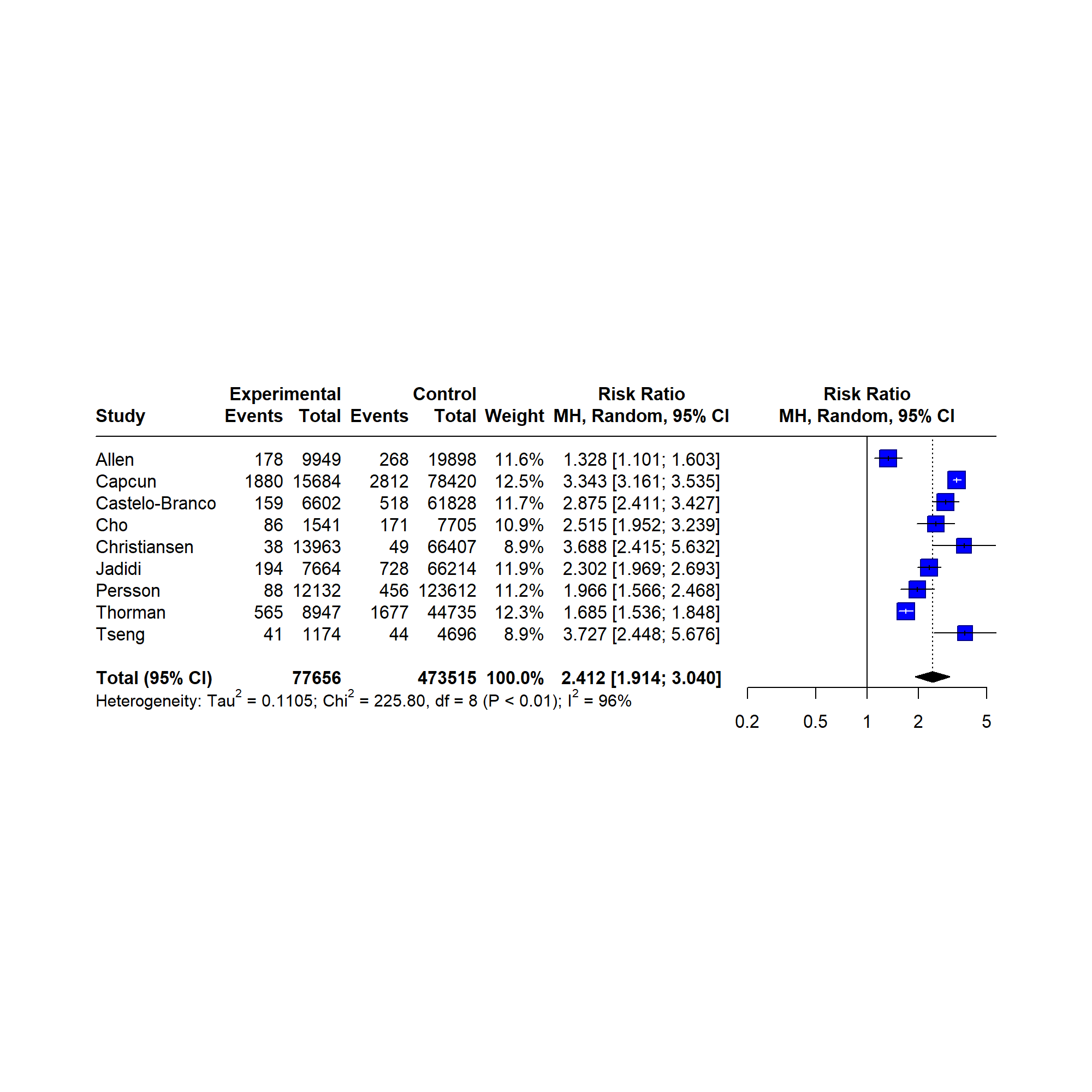


Supplementary Figure 8: Pooled prevalence of all-cause stroke in MS patients in sensitivity analysis after exclusion of studies with duration shorter than 10 years


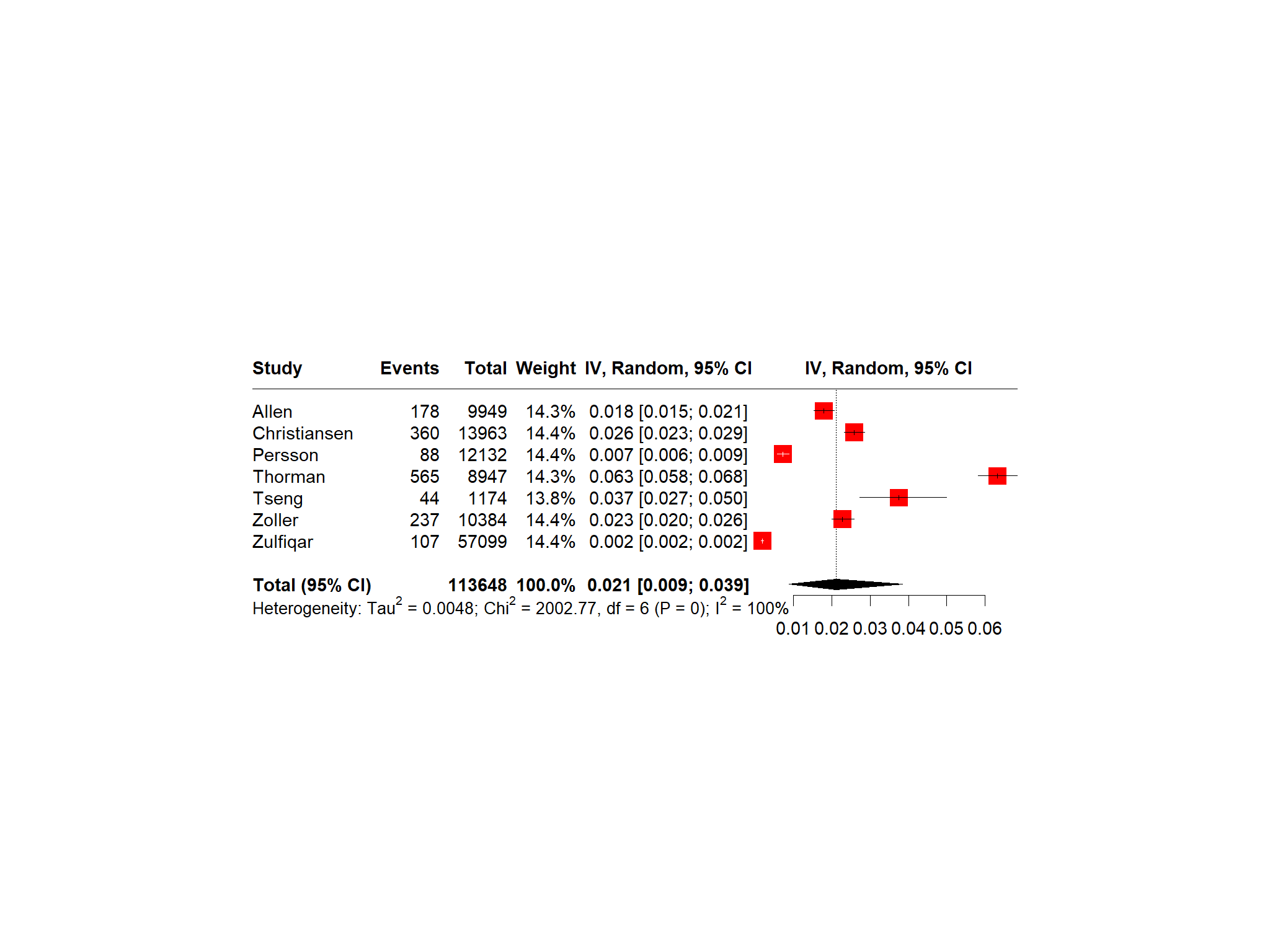


Supplementary Figure 9: Relative risk for all-cause stroke in the MS population compared to the general population in sensitivity analysis after exclusion of studies with duration shorter than 10 years


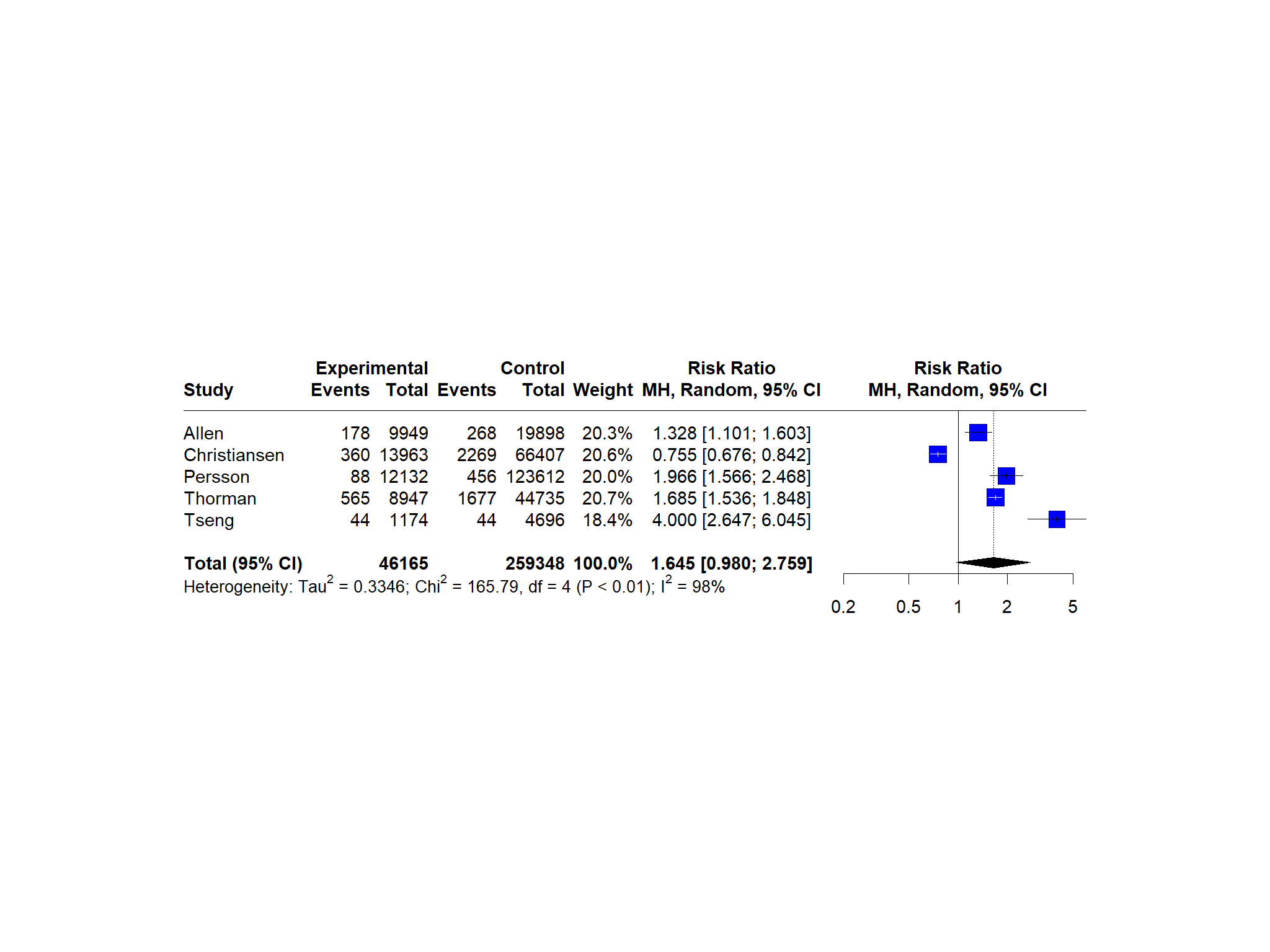


Supplementary Figure 10: Funnel Plot of all-cause stroke prevalence in the MS population


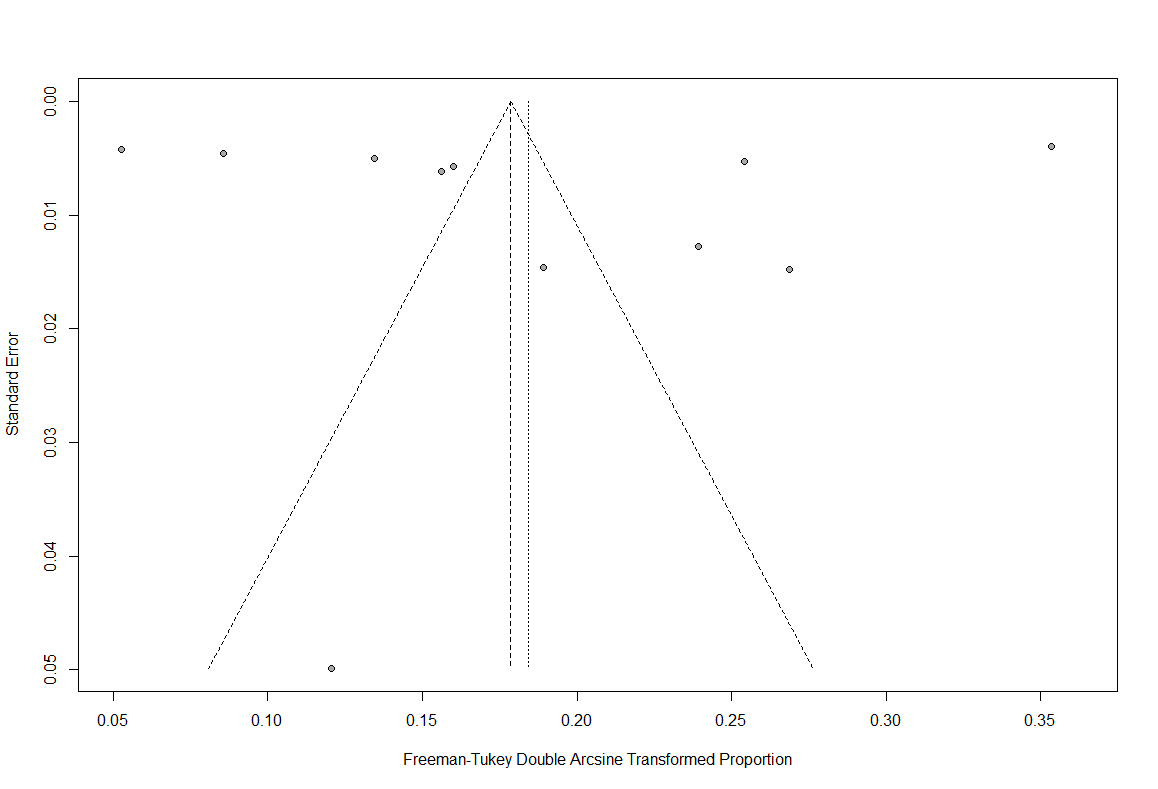


Supplementary Figure 11: Funnel Plot of the relative risk for all-cause stroke in the MS population compared to the general population


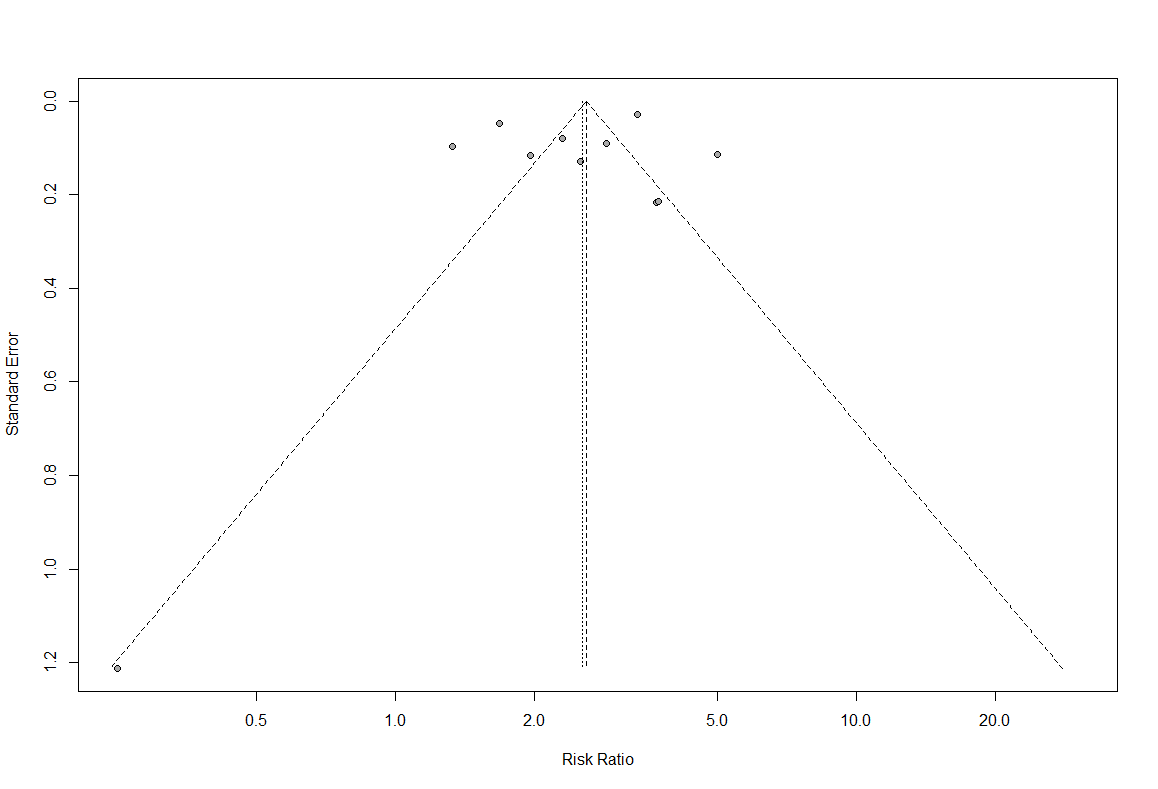

Supplement: Supplementary file 1 — Supplementary file1 (DOCX 616 KB) [file 415_2024_12331_MOESM1_ESM.docx]
